# A Study of the Relationship between Serum Bile Acids and Propranolol Pharmacokinetics and Pharmacodynamics in Patients with Liver Cirrhosis and in Healthy Controls

**DOI:** 10.1371/journal.pone.0097885

**Published:** 2014-06-06

**Authors:** Anne B. Taegtmeyer, Manuel Haschke, Lydia Tchambaz, Mirabel Buylaert, Martin Tschöpl, Ulrich Beuers, Jürgen Drewe, Stephan Krähenbühl

**Affiliations:** 1 Division of Clinical Pharmacology & Toxicology, University and University Hospital Basel, Basel, Switzerland; 2 Department of Biomedicine, University of Basel, Basel, Switzerland; 3 Department of Gastroenterology & Hepatology, Academic Medical Center, University of Amsterdam, Amsterdam, The Netherlands; University of Basque Country, Spain

## Abstract

The main objectives of the study were to determine the exposure and bioavailability of oral propranolol and to investigate their associations with serum bile acid concentration in patients with liver cirrhosis and in healthy controls. A further objective was to study the pharmacodynamics of propranolol. An open-label crossover study was performed to determine the pharmacokinetics and pharmacodynamics of propranolol after oral (40 mg) and intravenous (1 mg) administration as well as the concentration of total and individual fasting serum bile acids in 15 patients with liver cirrhosis and 5 healthy controls. After intravenous propranolol, patients showed a 1.8-fold increase in the area under the plasma concentration-time curve (AUC_0–∞_), a 1.8-fold increase in volume of distribution and a 3-fold increase in the elimination half-life (mean ± SEM: 641±100 vs. 205±43 minutes) compared to controls. After oral application, AUC_0–∞_ and elimination half-life of propranolol were increased 6- and 4-fold, respectively, and bioavailability 3-fold (83±8 vs. 27±9.2%). Maximal effects on blood pressure and heart rate occurred during the first 4 and first 2 hours, respectively, after intravenous and oral application in both patients and controls. Total serum bile acid concentrations were higher in patients than controls (42±11 vs. 2.7±0.3 µmol/L) and were linearly correlated with the serum chenodeoxycholic acid concentration. There was a linear correlation between the SBA concentration and propranolol oral AUC_0–∞_ in subjects not receiving interacting drugs (r^2^ = 0.73, n = 18). The bioavailability of and exposure to oral propranolol are increased in patients with cirrhosis. Fasting serum bile acid concentration may be helpful in predicting the exposure to oral propranolol in these patients.

## Introduction

The bioavailability and clearance of drugs primarily metabolized by the liver can be affected by liver cirrhosis [1]–[3]. This is especially true for drugs which have a high hepatic extraction (high-extraction drugs) [1], [4]. Per definition, high-extraction drugs are eliminated by more than 60% during the first passage across the liver, resulting in an oral bioavailability of less than 40% in healthy subjects. In patients with liver cirrhosis, the bioavailability of such drugs can reach 100% due to intra- and extrahepatic porto-systemic shunts [1], [5], [6]. High-extraction drugs must therefore be dosed very carefully in this population to avoid dose-dependent adverse reactions [7].

The clearance of high-extraction drugs, which is grossly determined by blood (or plasma) flow across the liver [1]–[3], may also be reduced in cirrhosis as a result of impaired blood flow [8], [9]. In contrast to increased porto-systemic shunting which only affects the pharmacokinetics of orally administered drugs, impaired drug clearance affects the pharmacokinetics of both orally and intravenously administered drugs and can prolong the exposure to high drug concentrations, thereby increasing the risk of toxicity.

Propranolol is a high-extraction drug [10], which is used frequently in patients with cirrhosis to prevent variceal bleeding [11]–[13] as it reduces hepatic blood flow and portal pressure [14], [15]. As expected, the pharmacokinetic properties of propranolol are altered in patients with cirrhosis compared to normal subjects. After intravenous (iv) application, the half-life of propranolol is increased due to an increase in the volume of distribution and a decrease in hepatic clearance [15], [16]. After oral administration, the exposure to propranolol is much higher in patients with cirrhosis compared to patients without liver disease, [17], [18] suggesting an increase in bioavailability in addition to impaired clearance. Clinically it is well established that propranolol has to be started at very low doses and that careful up-titration is necessary to find the appropriate dose for individual patients, especially in patients with Child class C cirrhosis [12], [14]. While the Child-Pugh score is often used to guide dosing in cirrhosis, this score is only validated for assessment of prognosis in patients with liver cirrhosis and does not reflect pharmacokinetic or pharmacodynamic properties of drugs in these patients [1].

The extent of porto-systemic shunting appears to be a main determinant of exposure to orally administered high-extraction drugs such as propranolol [1], [15]. Considering this observation, we reasoned that a correlation between markers of porto-systemic shunting such as fasting serum bile acids (SBA) and exposure to and/or bioavailability of propranolol in patients with cirrhosis might exist. Bile acids are almost completely extracted by the liver [19], [20] and have been shown to correlate linearly with the magnitude of the porto-systemic shunt [21]. We therefore hypothesized that fasting SBA concentrations can be used to predict the exposure to and/or bioavailability of orally administered high-extraction drugs such as propranolol. Accurate prediction of the bioavailability and exposure of high-extraction drugs such as propranolol prior to initiating therapy could help in determining the most effective and safest initial dose for patients with liver cirrhosis.

The specific aims of the study were to determine the kinetics - including absolute bioavailability - of propranolol after oral and iv application in healthy subjects and patients with liver cirrhosis and to correlate pharmacokinetic parameters with the serum concentrations of total and individual bile acids. A further aim was to investigate the relationship between propranolol pharmacokinetics and pharmacodynamics. The study aims could be achieved.

## Materials and Methods

### Ethics Statement

Written informed consent was obtained from each patient and healthy control subject. The study protocol conformed to the ethical guidelines of the 1975 Declaration of Helsinki as reflected in *a priori* approval by the institutional review committee, Ethikkommission Beider Basel (Protocol number 141/01, approved 27^th^ July 2001).

### Study Design

**Figure 1:**
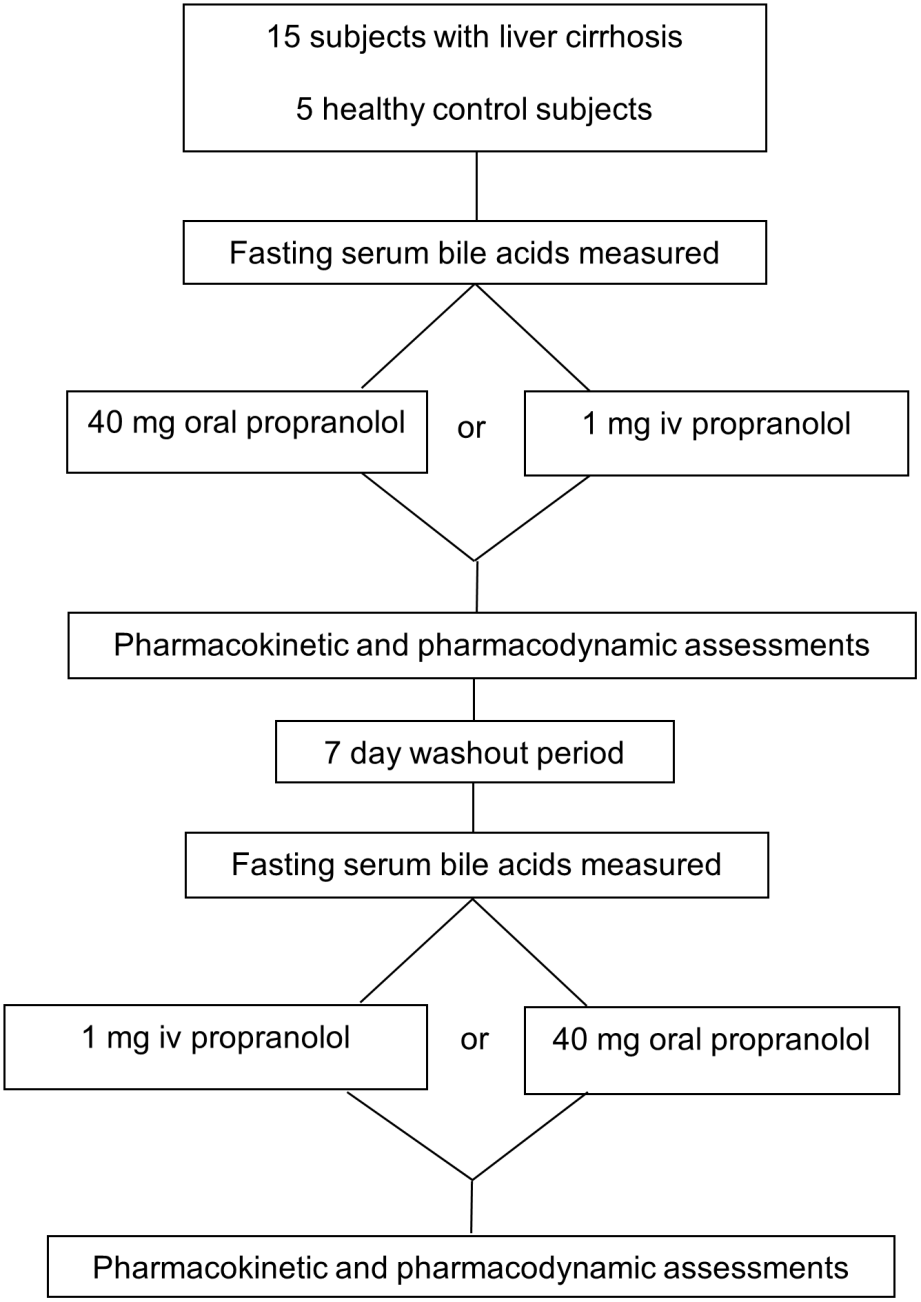
Study flow chart.

The study had an open cross-over design and consisted of two individual sessions (Figure 1). Study subjects were randomly assigned to receive either 1 mg propranolol given as an iv infusion over 10 minutes using a perfusor pump (Inderal, AstraZeneca AG, Zug, Switzerland, 1 mg/1 ml ampoule dissolved in 50 ml 0.9% sodium chloride solution) or 40 mg propranolol given as an oral tablet (Inderal, AstraZeneca AG Zug, Switzerland) in the fasted state. Allocation to treatment was performed according to a randomization list using random number generation. Pharmacokinetic sampling was performed and pharmacodynamic assessments were made before, during and after each session. Pharmacodynamic assessments consisted of non-invasive blood pressure and heart rate monitoring. Eight patients and three controls also underwent superior mesenteric artery and portal vein blood flow measurements. After a minimum seven day wash-out period, subjects received the other formulation of propranolol in a second session and underwent the same assessments as previously. An end-of-study visit was carried out two to 10 days after the last propranolol administration.

### Subjects

Five healthy individuals (the control group) and 15 patients with liver cirrhosis were studied between October 2002 and August 2004. Study physicians enrolled participants into the study. Since no *a priori* data were available on the variability of study parameters, the sample size was chosen on the basis of practical considerations. The control subjects had no evidence of liver disease, as assessed by medical history and physical examination. The 15 patients with liver cirrhosis were recruited from outpatients regularly seen at the Hepatology Unit of the University Hospital of Basel. Patient inclusion criteria were age between 20 and 70 years, liver cirrhosis as verified by liver biopsy and/or by typical clinical and sonographic signs such as skin signs of chronic liver disease, enlarged spleen and ascites. Exclusion criteria are listed in the on-line supplement. Demographics and characteristics of the patients with liver cirrhosis and the control subjects are given in Table 1. Child and Meld scores were calculated according to the original publications [22]–[24]. Patients usually receiving propranolol were instructed to stop taking it one week before the start of the study and to restart it after the last pharmacokinetic study day.

**Table 1:** Patient and control demographics.

Subject	Age(years)	Sex	BMI(kg/m^2^)	Underlyingcondition	Child score(class)	Meldscore	Albumin(g/L)	AP(U/L)	Bilirubin(µmol/l)	Cholic acid(µmol/l)	Chenod-eoxycholic acid(µmol/l)	Total serumbile acids(µmol/l)	Shuntindex (%)
Patient 1	57	M	20.4	ALD andHepatitis B	7 (B)	11	35	142	46	1.7	5.3	6.0	7
Patient 2	51	F	31.2	Hepatitis C	6 (A)	7	35	79	9	4.3	19.2	25.3	21
Patient 3¥	27	M	20.0	Hepatitis B	9 (B)	26	28	124	183	32.9	112.6	167	
Patient 4	54	M	25.1	ALD	12 (C)	27	17	327	178	8.2	42.4	30.4	24
Patient 5	47	F	21.2	Hepatitis C	6 (A)	10	31	167	18	2.6	11.8	12.7	12
Patient 6	37	m	24.2	Hepatitis C	6 (A)	9	35	102	11	1.8	4.1	8.5	9
Patient 7†	50	m	25.4	ALD	12 (C)	6	28	235	133	26	77.4	87.7	64
Patient 8†	49	f	28.1	NASH	9 (B)	24	24	28	44	18.2	70.2	80.8	59
Patient 9	50	m	29.6	ALD	11 (C)	14	27	314	393	5.9	53.3	40.1	31
Patient 10	60	m	31.8	ALD	6 (A)	21	35	261	29	11.8	22.8	18.5	16
Patient 11	60	m	30.4	ALD	6 (A)	10	31	104	20	8.5	15.6	29.7	24
Patient 12	51	m	20.4	ALD	9 (B)	10	25	286	117	7.1	65.1	57.7	43
Patient 13†	58	m	21.3	ALD	8 (B)	14	24	50	8	5.4	10.1	27.1	22
Patient 14	67	m	26.7	Hepatitis C	5 (A)	7	36	101	17	3.6	10.9	13.8	13
Patient 15	50	m	23.5	ALD	7 (B)	12	32	95	43	4	9	22.8+	19
**Summary patients**	Median 51	80% m	25.3±1.1		Median 6	Median 11	30±1	161±24	83±27	7.8±1.8*	30±7*	42±11*	26±5*
Control 1	23	m	20.9	None						0.9	2.0	3.8	6
Control 2	26	f	23.3	None						1.5	1.6	2.7	5
Control 3	21	m	24.1	None						1.2	2.0	1.7	4
Control 4	62	m	25.7	None						1.3	1.2	2.9	5
Control 5	51	f	23.5	None						1.2	0.8	2.5+	5
**Summary controls**	Median 26	60% m	23.5±0.8							1.2±0.1	1.5±0.3	2.7±0.3	5±0.3

Abbreviations: ALD = alcohol associated liver disease, AP = alkaline phosphatase, NASH = non alcoholic steatohepatitis, m = male, f = female, y = yes, n = no, uk = unknown. Data are summarised as mean ± standard error of the mean unless otherwise stated. All cases of cirrhosis were confirmed on histology excluding patient 8 and patient 12.

¥ Case excluded from bile acid summary statistics and calculation of shunt index due to greatly elevated serum bile acids under concomitant treatment with cyclosporine (see main body of text for mechanistic explanation).

*Two-sided unpaired t-test on log-transformed data p<0.005 vs. control.

+ sum of the individually determined bile acids (see Figure S1C in File S1 for correlation).

† previous therapy with propranolol.

### Pharmacokinetic Sampling and Pharmacodynamic Measurements

After propranolol administration, blood sampling was performed through a designated indwelling forearm catheter placed, if necessary, in a vein of the contralateral arm to the catheter used for drug administration. Venous blood samples (5 ml) were collected into heparinized tubes, centrifuged at 3000 revolutions per minute for 10 minutes and the supernatant stored at −20°C until further analysis. Blood samples were collected at 5 minutes, 0.25, 0.5, 1, 1.5, 2, 3, 5, 8, 14, 24 and 48 hours (h) after propranolol dosing. After iv application additional samples were collected at 10 minutes and 0.75 h. Non-invasive blood pressure and heart rate were determined in the lying position at the same time points as blood sampling.

Superior mesenteric artery and portal venous blood flow were measured by the Duplex technique consisting of a real-time section scanner (3.5 MHz) and a pulsed Doppler flow meter (3.5 MHz) (Hitachi Medical Systems, formerly Aloka). The details of the technique employed to measure blood flows have been published previously [25]. Superior mesenteric artery and portal venous blood flow measurements were made just before propranolol administration and at 10 and 90 minutes after dosing.

### Propranolol Measurement

Propranolol plasma levels were measured using a high pressure liquid chromatography (HPLC)-assay. A simple method was developed to determine low propranolol concentrations in heparinized human plasma using pronethalol as an internal standard. Details of the assay are given in the online supplement.

### Serum Bile Acid Measurement

The total serum bile acid pool was determined in fasting serum samples obtained immediately prior to each propranolol administration using a commercially available enzymatic spectrophotometric assay (TOTAL BILE ACIDS-HR, Enzymatic method, Wako, Osaka, Japan). For the purpose of subsequent calculations, the mean of the two baseline total SBA measurements was employed. As bile acid measurement took place prior to propranolol dosing, measurements were performed on samples taken at least 7 days apart from one another.

The serum concentrations of the individual bile acids were determined by gas chromatography mass spectroscopy as described previously [26]. Conventional liver function tests, other biochemical determinations and haematological investigations were performed using standard methods in the Departments of Clinical Chemistry and Haematology of the University Hospital of Basel.

The shunt index was estimated using the serum bile acid concentration according the equation published by Ohkuba and colleagues [21]:
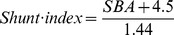



### Pharmacokinetic Calculations

Analysis of the plasma samples provided two individual plasma concentration-time curves for each subject. Values below the limit of quantification (0.5 ng/ml) were set to zero for use in calculations. The area under the plasma concentration-time curves (AUC) was obtained by the trapezoid rule with linear interpolation using a non-compartmental model (PK-Solver) [27]. Bioavailability, clearance, half-life and apparent volume of distribution were calculated according to the standard pharmacokinetic equations given in the online supplement.

### Statistical Analysis

Summary data are expressed as mean and standard error of the mean. Group means were compared by two-tailed unpaired t-test on log-transformed data where appropriate. Correlations were assessed using linear regression analysis or the Spearman rank test. The level of significance was p = 0.05. All statistical analyses were performed with STATA (STATA version 9, College Station, USA).

## Results

### Study Population

Study subject characteristics are given in Table 1. Patients were older than control subjects (not statistically significant) and had a slightly higher body mass index. Most prevalent reasons for liver cirrhosis were alcoholic liver disease and infectious hepatitis. Six patients were in Child-Pugh class A, 6 in B and 3 in C. In comparison to control subjects, patients had significantly higher total serum bile acid concentrations as well as increased serum concentrations of the main bile acids chenodeoxycholic acid and cholic acid (Table 1). There was no overlap for these analytes between patients and control subjects. Two control subjects and seven patients did not undergo doppler assessment due to staffing constraints.

### Pharmacokinetics

**Figure 2:**
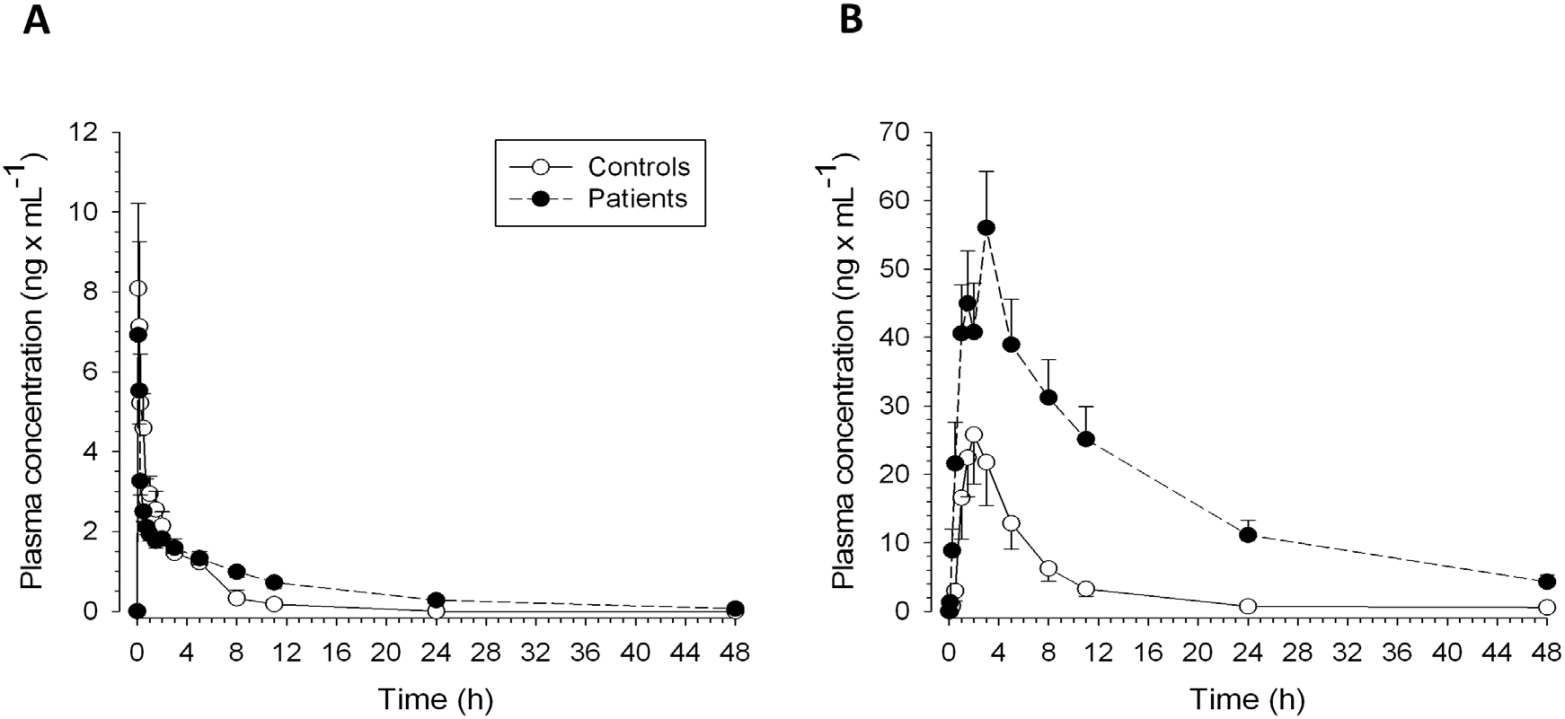
Propranolol plasma concentration versus time profiles after (A) intravenous (1 mg) and (B) oral (40 mg) application. Plasma concentrations were determined using high-performance liquid chromatography (HPLC). See Table 2 for calculated pharmacokinetic variables.

The pharmacokinetics of propranolol are shown in Table 2 and Figure 2. After iv application, the plasma concentration-time curves were similar between patients and control subjects. Accordingly, time until maximum concentration (T_max_), the maximum concentration (C_max_), clearance and AUC were not statistically different between the two groups. Patients had a significantly longer terminal elimination half-life compared to control subjects, which was explained by a significantly increased V_d_ and a numerically decreased clearance of propranolol. The AUC_0–∞_ was approximately doubled in patients. After oral application, patients had an approximately 6-fold greater AUC compared to control subjects (p = 0.009) and the mean C_max_ was more than double that seen in the control group. Patients also showed impaired elimination compared to controls; the elimination half-life was approximately 4-fold longer (p = 0.002). The mean bioavailability of propranolol was 83% in patients and 27% in control subjects (p = 0.002).

**Table 2:** Propranolol pharmacokinetic data.

Subject	Intravenous							Oral					
	Dose	C_max_	T_max_	AUC_0–∞_	Cl	V_d_	T_1/2_	Dose	C_max_	T_max_	AUC_0–∞_	T_1/2_	F (%)
	(mg)	(ng×ml^−1^)	(min)	(ng×min ×ml^−1^)	(ml×min^−1^)	(l×kg^−1^)	(min)	(mg)	(ng×ml^−1^)	(min)	(ng×min×ml^−1^)	(min)	
Patient 1	1.0	2.3	10	889	1125	8.1	293	40	90	120	39289	346	111
Patient 2	1.0	18.7	5	1818	550	5.0	477	40	59	90	48787	800	67
Patient 3	1.0	10.0	10	1310	763	5.0	257	40	52	480	55299	627	106
Patient 4†	1.0	2.4	10	3029	330	5.2	824	40	156	180	143176	711	118
Patient 5	1.0	6.3	10	883	1133	13.4	485	40	25	90	22707	958	64
Patient 6	1.0	12.1	5	791	1265	9.3	358	40	50	90	14143	346	45
Patient 7	1.0	7.0	5	1752	571	4.9	504	40	63	180	72859	1029	104
Patient 8	1.0	7.4	5	7200	139	4.8	1718	40	62	180	88124	1237	31
Patient 9	1.0	2.2	10	1264	791	7.2	569	40	33	300	49476	1096	98
Patient 10	1.0	8.4	5	1432	698	10.0	934	40	20	60	17894	1362	31
Patient 11	1.0	10.6	5	1536	651	9.1	888	40	132	180	76244	838	124
Patient 12	1.2	5.9	10	1964¥	606¥	11.6	913	40	32	60	66527	1643	84
Patient 13	1.0	2.2	5	2540	394	8.2	934	40	53	180	42261	523	42
Patient 14	1.0	11.8	5	1004	996	4.9	275	40	82	60	44270	801	110
Patient 15	1.0	7.3	5	505	1981	8.2	193	40	59	60	23729	342	117
**Summary statistics**		7.6±1.1	5	1778±441	833±120	7.7±0.7*	641±100**		65±9	120	47260±5900*	844±96*	83±8*
Control 1	1.0	7.2	10	785	1273	5.7	230	40	9	90	1332	85	4
Control 2	1.0	8.9	5	1469	681	2.6	198	40	22	180	8035	165	14
Control 3	1.0	14.1	5	856	1169	4.1	158	40	60	120	15816	176	46
Control 4	1.0	13.3	5	491	2038	3.4	90	40	30	180	9978	334	51
Control 5	1.0	18.1	5	1295	772	6.1	347	40	21	180	9471	310	18
**Summary statistics**		12.3±2	5	979±177	1187±241	4.4±0.7	205±43		28±9	180	8930±2320	214±47	27±9.2

Abbreviations: AUC = area under the concentration-time curve; Cl = clearance; C_max_ = peak concentration, C_min_ = trough concentration, F = oral bioavailability, T_1/2_ = elimination half-life; T_max_ = time point of C_max_; V_d_ = volume of distribution, NA = not available. Data are summarised as mean ± standard error of the mean or median as appropriate.

† Case excluded from AUC and Cl statistics due to the effect of concomitant treatment with ciprofloxacin (see main body of text for mechanistic explanation),

*p<0.01 vs. control subjects,

**p<0.05 vs. control subjects,

¥ corrected for dose.

### Correlation of SBA with Propranolol Pharmacokinetics

The reliability of the serum bile acid determination was confirmed by comparing the measurements obtained before the administration of both the oral and the iv propranolol. As shown in Figure S1A in File S1, there was a linear correlation between these two samples (y = 8.1+0.74x, r^2^ = 0.865). Accordingly, the calculations were performed with the average value of the two samples. In addition, there was also a linear correlation between the SBA concentration with the serum concentration of chenodeoxycholic acid, cholic acid and the total of the individually measured serum bile acids (Figures S1B–D in File S1).

**Figure 3:**
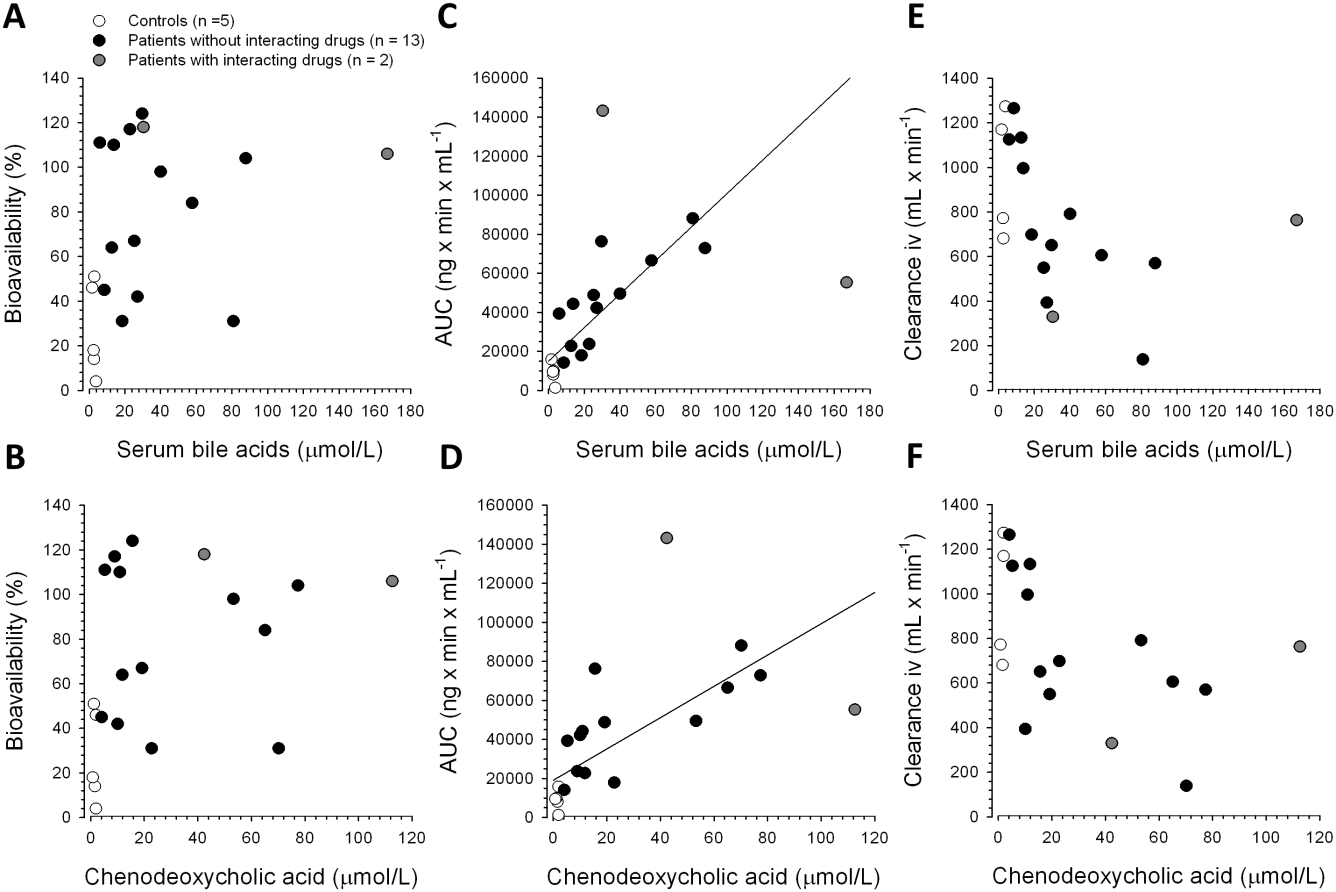
Relationship between the serum bile acid or chenodeoxycholic acid concentration with bioavailability (A,B), propranolol AUC after oral application (C,D), and clearance after intravenous application (E,F).

As shown in Figures 3A and B, there was no significant linear correlation between SBA or chenodeoxycholic acid concentrations and propranolol bioavailability. In contrast, there was a significant linear correlation between the SBA and the AUC_0–∞_ of oral propranolol in all subjects (y = 14961+859x, r^2^ = 0.73) and in patients alone (y = 23095+710x, r^2^ = 0.63) (Figure 3C). The relationship between chenodeoxycholic acid concentrations and the AUC_0–∞_ of oral propranolol in all subjects and in patients alone could be described by the following two equations: y = 19132+802x, r^2^ = 0.62 and y = 28625+624x, r^2^ = 0.51, respectively (Figure 3D). There was a weak inverse correlation between the SBA and propranolol clearance after iv administration in patients and controls (y = 1193–10.4x, r^2^ = 0.3) (Figure 3E). Similarly, there was a weak inverse correlation between serum chenodeoxycholic acid concentration and clearance after administration of iv propranolol (y = 1155–10.4x, r^2^ = 0.29 in all subjects, and y = 1116–9.6x, r^2^ = 0.31 in patients alone) (Figure 3F).

Two patients (shown in grey in Figure 3) were excluded from these analyses because they were retrospectively found to have been receiving interacting drugs. Patient 3 (Tables 1 and 2) was a renal allograft recipient under immunosuppression with cyclosporine and had very high fasting SBA. Cyclosporine inhibits the bile salt export pump responsible for canalicular transport of bile acids into bile [28] so the elevated SBA in this patient was only partially related to porto-systemic shunting. Patient 4 received concomitant ciprofloxacin. Ciprofloxacin is a strong inhibitor of CYP1A2 [29] which is important for propranolol metabolism [30]. The very high exposure to propranolol seen in Patient 4 was therefore not solely related to porto-systemic shunting (though this is likely to have further increased exposure in the presence of CYP 1A2 inhibition).

In addition, there were significant rank correlations between total serum bile acid, chenodeoxycholic acid serum concentrations and shunt index and pharmacokinetic and prognostic parameters (Table 3; patients 3 and 4 were excluded from these analyses). In particular SBA concentrations correlated well with AUC_0–∞_ and half-life of propranolol after oral application (Table 3). Child class was only weakly associated with AUC after oral administration (Spearman’s rho 0.56, p = 0.05) and was not associated with bioavailability (Spearman’s rho 0.21, p = 0.48). Meld score did not correlate with AUC after oral administration, nor did it correlate with SBA.

**Table 3:** Correlation between total serum bile acids, chenodeoxycholic acid and shunt index and pharmacokinetic and prognostic parameters.

	Total serum bile acids		Chenodeoxycholic acid		Shunt index	
Parameter	Spearman’s rho	p	Spearman’s rho	p	Spearman’s rho	P
AUC_0–∞_ (ng.min/ml) iv	0.64	0.004	0.62	0.006	0.66	0.003
Clearance (ml/min) iv	−0.64	0.004	−0.62	0.006	−0.66	0.003
Half-life iv (min)	0.76	0.0003	0.77	0.0002	0.77	0.0002
AUC_0–∞_ (ng.min/ml) po	0.89	<0.0001	0.86	<0.0001	0.89	<0.0001
Half-life po (min)	0.82	<0.0001	0.91	<0.0001	0.82	<0.0001
Bioavailability (%)	0.43	0.08	0.38	0.12	0.41	0.09
Child score	0.71	0.006	0.54	0.05	0.71	0.006
Meld Score	0.09	0.77	0.06	0.9	0.09	0.77

Correlations with pharmacokinetic parameters were performed in patients (n = 13) and controls (n = 5) while correlation with prognostic factors were only performed in patients (n = 13). Patients 3 and 4 excluded because of interacting medication (see text). AUC_0–∞_ = area under the curve time zero to infinity, iv = intravenous, po = oral administration.

### Pharmacokinetic-pharmacodynamic Relationships

**Figure 4:**
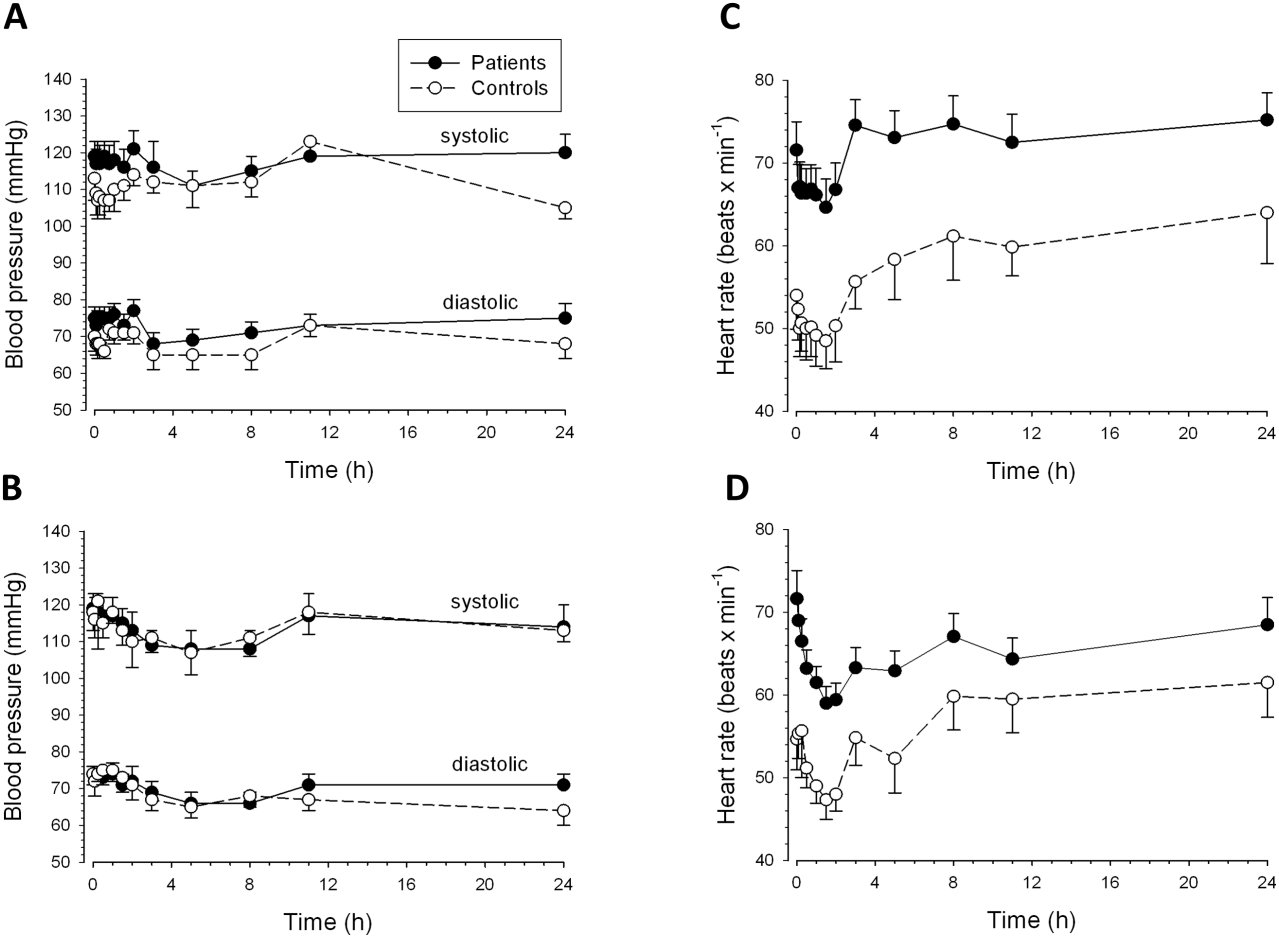
Effect of propranolol on non-invasive hemodynamic variables. Blood pressure vs. time curves after (A) intravenous (1 mg); and (B) oral (40 mg) application and heart rate vs. time curves after (C) intravenous (1 mg) and (D) oral (40 mg) application of propranolol. The values at 48 h are not shown since they were not significantly different from those at 24 h.

The effects of propranolol on blood pressure and heart rate are shown in Figure 4. Basal blood pressure before propranolol was similar in patients and control subjects. Oral and iv propranolol decreased systolic and diastolic blood pressure by 8 to 12 mmHg in both patients and controls. The maximal effect was reached during the first 4 h after application and the blood pressure reached basal values again after approximately 12 h (Figures 4A and B). Patients had a higher basal heart rate than control subjects (Figures 4C and D). After iv propranolol, the heart rate decreased by approximately 5 beats/min in controls and by approximately 7 beats/min in patients (Figure 4C). After oral administration, the corresponding figures were approximately 7 beats/min in controls and 12 beats/min in patients (Figure 4D). The maximal effect was reached after 1 to 2 h in both groups and basal or exceeding values were reached after 3 h (iv administration) or after 8 to 24 h (oral ingestion).

**Figure 5:**
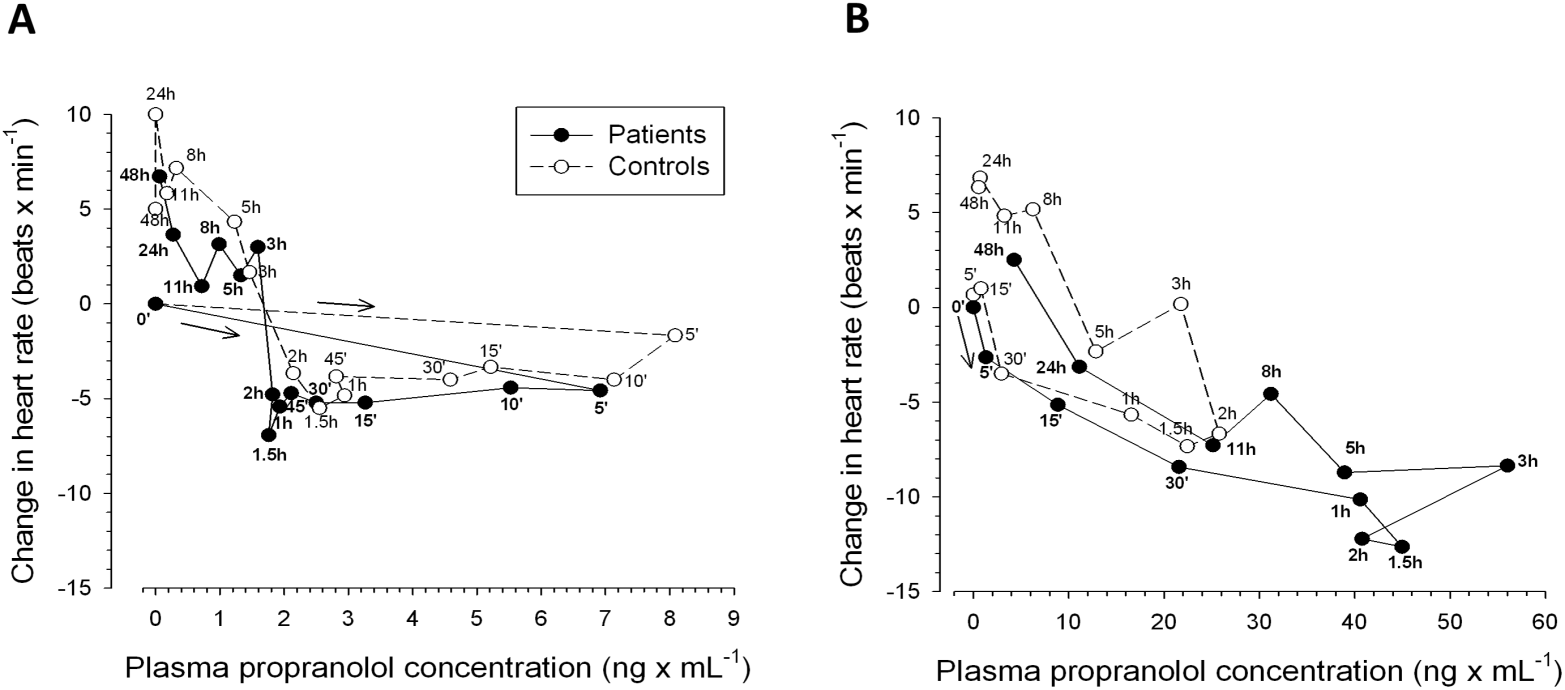
Relationship between mean propranolol plasma concentrations and change in heart rate after (A) intravenous (1 mg) and (B) oral (40 mg) administration of propranolol.

The correlation between the propranolol plasma levels and effect on heart rate showed that the pharmacodynamic action of propranolol was almost identical in patients and control subjects (Figure 5). After iv application, there was a rapid effect (within 5 minutes) on the heart rate. This effect became more pronounced during the time period when plasma levels were already decreasing and reached a maximum at 1.5 h after dosing. At 3 h and beyond the heart rate exceeded the basal rate (Figures 4C and 5A). The hysteresis was small and clock-wise, reflecting rapid equilibration between plasma and target site (β_1_-receptors) as well as persistence of the pharmacological effect when plasma concentrations are decreasing (Figure 5A). After oral ingestion, the heart rate started to decrease after 5 minutes in patients and after 30 minutes in control subjects (Figure 4D and Figure 5B). The maximal effect was dependent on the plasma concentration and was reached at 1.5 to 2 h in both patients and control subjects. The hysteresis was again small but, in contrast to iv application counter clock-wise, reflecting decreasing pharmacological effects when the plasma concentrations are still increasing (Figure 5B).

### Correlation of SBA with Propranolol Pharmacodynamics

Maximum change in systolic blood pressure and heart rate after oral propranolol administration are shown in Table S1 in File S2. Patients showed a significantly greater fall in heart rate (p = 0.03) but not in systolic blood pressure compared to controls. However there were no significant linear correlations between changes in systolic blood pressure and heart rate and total serum bile acid concentration (data not shown).

### Liver Blood Flow

As shown in Table S2 in File S2 and Figure S2 in File S1, propranolol affected the blood flow in both the superior mesenteric artery and the portal vein. Patients had a higher basal blood flow than controls in both the superior mesenteric artery and the portal vein. Compared to basal values, iv and oral propranolol decreased the blood flow in the superior mesenteric artery by 15 to 20% in both patients and control subjects. Compared to basal values, propranolol reduced the portal vein blood flow in patients after iv and oral application by 4 and 13%, respectively. In controls, the corresponding figures were 9 and 34%.

### Adverse Events

Adverse events were one case of an asymptomatic fall in systolic blood pressure of 40 mmHg 15 minutes after dosing whilst supine and mild dizziness on exertion which were assessed as having a probable association with the administration of oral propranolol (patient 4), transient loss of appetite (patient 5, unrelated) and asymptomatic blood pressure elevation 24 hours after iv propranolol (patient 14, unrelated). No adverse events were experienced by control subjects.

## Discussion

In the current study of propranolol pharmacokinetics after iv and oral administration in patients with liver cirrhosis and healthy controls there was a linear correlation between fasting SBA concentration and propranolol exposure. The study findings also confirmed those reported in previous studies, namely that clearance after intravenous administration is reduced [16], plasma concentrations after oral administration are higher [17], [18] and that the heart rate response to oral propranolol is greater in patients than controls [18]. We were also able to further characterize propranolol pharmacokinetic-pharmacodynamic relationships in patients with liver cirrhosis.

Taking into account that there is a constant flow of bile acids into the duodenum which is independent of food-intake (also in humans with an intact gallbladder) [19], [31], the serum bile acid concentration in fasting individuals is determined both by the quantity of bile acids reaching the systemic circulation and bile acid clearance. In healthy individuals, the quantity of bile acids reaching the systemic circulation is very small because of the liver’s ability to extract and clear bile acids from portal blood. In comparison, the quantity of bile acids reaching the systemic circulation is higher in patients with liver cirrhosis due to porto-systemic shunts and a reduced hepatic capacity to clear bile acids during their first passage across the liver. In healthy individuals, SBA clearance is mainly limited by hepatic blood flow [19]. In comparison, in patients with liver cirrhosis, not only hepatic blood flow but also hepatic handling of bile acids - in particular the uptake of bile acids by hepatocytes and/or canalicular export - may become rate-limiting. Hepatocellular uptake and export of bile acids are primary or secondary active transport processes [32]–[34] which may be impaired in patients with liver cirrhosis. The pharmacokinetic behaviour of propranolol is in many ways similar to that of bile acids. In healthy individuals, propranolol clearance – like SBA clearance - is also limited by liver blood flow [10], and in patients with cirrhosis exposure after oral ingestion is highly dependent on the presence of porto-systemic shunts [17], [18].

The increased bioavailability of propranolol in patients can mainly be explained by the presence porto-systemic shunts and decreased metabolism of propranolol during the first passage across the liver. Since the mean estimated shunt index was 26% (Table 1), it is likely that not only porto-systemic shunting but also decreased hepatic metabolism of propranolol contributed to the observed increase in bioavailability (from a mean value of 27% in controls to 83% in patients). In support of this assumption, the cytochrome P450 enzymes (CYPs) associated with the metabolism of propranolol (mainly CYP2D6, CYP1A2 and CYP2C19 [30], [35]) have all been shown to have a reduced protein content and/or activity in cirrhotic livers [36]–[38].

There was no correlation between the serum bile acid concentration and bioavailability of propranolol. This may largely be explained by the finding that impaired hepatic metabolism of propranolol is an important cause of the increase in propranolol bioavailability whereas the CYPs involved in propranolol metabolism do not play a role in the hepatic handling of bile acids.

Drug exposure after oral ingestion (reflected by AUC_0–∞_) was 6-fold greater in patients as compared with control subjects. The AUC_0–∞_ after oral ingestion of propranolol is a function of the amount of the dose reaching the systemic circulation and drug elimination. Since the propranolol dose reaching the systemic circulation is the product of bioavailability and ingested dose, the 6-fold increase in oral AUC_0–∞_ can partially be explained by increased bioavailability (increased by a factor of 3 in patients compared to controls; Table 2) and to an approximately equal part by impaired elimination. Drug elimination, as reflected by the elimination rate constant or half-life of propranolol, is a function of the ratio of drug clearance and volume of distribution. In comparison to controls, patients had a 30% percent decrease in propranolol clearance and nearly a doubling of the volume of distribution, leading to an approximately three-fold longer half-life (Table 2). As shown in Figure 3C, more than 60% of the variability of propranolol exposure could be explained by the variability in the SBA concentration, suggesting that the SBA concentration may be useful to predict propranolol exposure after the first oral dose.

The data in Figure 3C indicate that, for every 20 µM increase in SBA concentration, propranolol exposure increases roughly by the amount that would be expected at a SBA concentration of 0 (representing a patient with liver cirrhosis without porto-systemic shunts). Since the hemodynamic effects of propranolol are clearly dependent on its serum concentration (and therefore exposure) also in patients with liver cirrhosis (Figures 4 and 5), the first dose has to be chosen very carefully to avoid toxicity [12], [14].

The licensed initial dose of oral propranolol for the treatment of hypertension in patients with normal liver function in Switzerland is 80 mg q12 h. The licensed dose for angina pectoris is 40 mg q12 h – q8 h daily [13]. The US product information recommends 40 mg q12 h for hypertension and 80 mg daily as initial doses for hypertension and angina pectoris, respectively [39]. The licensed initial dose for the treatment of portal hypertension in Switzerland is 80 mg q24 h of a long acting propranolol preparation [13]. The dose is then titrated until a 25% reduction in heart rate from baseline is reached. Propranolol is not licensed for the treatment of portal hypertension in the USA [39], however recommendations from the Department of Veterans Affairs Hepatitis C Resource Center Program and the National Hepatitis C Program are to commence oral propranolol at 20 mg q12 h and to increase the dose as tolerated or to achieve a resting heart rate of 55–60 bpm [12]. In our study, one of 11 patients who neither had a durg-drug interaction which increased propranolol exposure (as did patient 4) nor had previously been treated with propranolol (as patients 7, 8 and 13 had been) experienced a greater than 25% drop in heart rate from baseline (Table S1 in File S2) and four had a heart-rate of less than 55 bpm after oral dosing. Approximately 1/3 of patients were therefore ‘overdosed’ according to the US-guidelines after receiving 40 mg oral propranolol.

The current drug label for the treatment of portal hypertension in Switzerland (80 mg/d) is too high in our opinion [13]. Taking the current findings together, we propose a propranolol starting-dose of 10 mg q12 h in patients with liver cirrhosis and a SBA concentration ≤20 µmol/L, and 10 mg q24 h in those with SBA concentrations >20 µmol/L. The individual maintenance dose could then be found by careful up-titration [12]–[14]. This could be tested against the currently labelled 80 mg in a randomised trial. Our proposed starting dose of 10 mg q12 h is in keeping with the study design and findings of Benares and colleagues’ randomized controlled trial of carvedilol vs. propranolol in reducing portal pressures in patients with cirrhosis [14]. The initial propranolol starting dose of 10 mg q12 h in this study could not be tolerated by all patients and at least one of the 25 patients randomised to receive propranolol required a dose-reduction to 10 mg per day [14]. All 3 patients in whom the study had to be discontinued due to an adverse event were in Child class B or C. Similarly Gonzalez-Abraldes and colleagues randomized 15 patients with cirrhosis to receive propranolol 20 mg twice daily, with subsequent dose-adjustment according to haemodynamic response (no decrease in heart rate to below 55 bpm and/or systolic blood pressure to below 90 mm Hg) and found that at least one patient subsequently required a maintenance dose of only 2.5 mg per day [40].

As shown in Figure S3 in File S1, the AUC after an oral dose in patients with cirrhosis may correlate better with SBA concentrations categorized into <20 µmol/L, 20.1–40 µmol/L and >40 µmol/L than with Child class, which is traditionally used to guide dosing of hepatically-eliminated drugs in cirrhosis. Dosing according to Child Class is however not labelled for propranolol [13], [39].

Non-parametric testing revealed close relationships between serum bile acid concentrations and variables of propranolol pharmacokinetics as well as the Child score. Similar correlations have also been described in previous studies of drug-disposition in patients with cirrhosis [41]. Since they could not be described by a linear mathematical function (as was the case for SBA concentration), however, they cannot be used to extrapolate dosing recommendations for propranolol in patients with liver cirrhosis.

Both patients and controls responded to propranolol with a decrease in heart rate and systolic and diastolic blood pressure. These are expected pharmacological effects of propranolol, a competitive non-selective β-adrenergic receptor antagonist. Propranolol inhibits sympathetic stimulation of the myocardium by competing with neurotransmitters such as catecholamines for binding at β1-adrenergic receptors. This leads to a reduction in resting heart rate, cardiac output, systolic and diastolic blood pressure in a dose- or concentration-dependent manner [13]. There was a close relationship between propranolol plasma concentrations and the pharmacodynamic action of propranolol in terms of heart rate and blood pressure, both in patients and in control subjects (Figures 4 and 5). The pharmacodynamic responses to intravenous propranolol were comparable between patients and controls, reflecting the similar propranolol concentrations in the two groups. Heart rate, but not blood pressure, was significantly more reduced in patients than controls after oral propranolol. This was likely due to higher propranolol exposure among patients. Interestingly, the plasma concentration-heart rate relationship showed a small clockwise hysteresis after iv application and a small counter-clockwise hysteresis after oral application both in control subjects and cirrhotic patients. After oral ingestion, the counter clock-wise hysteresis may be explained by the production of inactive metabolites which can interfere in the interaction of propranolol with the β1-adrenoreceptors [10]. Since metabolite production is more accentuated after oral than after iv dosing [10], an attenuated response even at high parent drug concentrations is possible.

The study did not find a significant correlation between change in heart rate or systolic blood pressure from baseline and SBA concentration. This may be due to the fact that 3 patients had previously been treated with propranolol. A larger, less heterogeneous patient group would need to be studied to investigate this further.

The study has a number of limitations. The small sample size precluded subgroup analyses, the aetiology of liver cirrhosis was not uniform across the patient group and the inclusion criteria were retrospectively found to be too wide. This latter point resulted in the inclusion of two patients whose data could not be used for determining the association between fasting bile acids and propranolol exposure after oral administration. However during the study planning and recruitment period, the effect of cyclosporine on the bile salt export pump and the CYP 1A2 inhibiting effects of ciprofloxacin were not known. Similarly, it cannot be ruled out that other currently unknown factors which affect serum bile concentrations or propranolol clearance may have been present in some individuals. Patients with cirrhosis are known to be particularly vulnerable to the effects of drug-drug interactions [42]. As the patients in this study represented a mixed group with differing underlying causes of liver cirrhosis, we believe, however, that the findings are generalisable to other patients with liver cirrhosis.

In conclusion, patients with liver cirrhosis had an increased exposure to propranolol after both iv and oral application, with exposure after oral application being six-fold higher than in healthy controls who received the same dose. This increase was explained by a greater bioavailability in the case of oral application as well as a decreased elimination of propranolol. There was a significant linear correlation between the serum bile acid concentration and propranolol exposure in patients without interacting co-medication. This correlation may be of use to clinicians in selecting the optimal initial dose of oral propranolol so that adverse reactions can be avoided. The clinical value of SBA-guided initial propranolol-dosing in patients with cirrhosis should be demonstrated in a prospective clinical study. Whether similar correlations between fasting SBA and drug-disposition exist for other high hepatic extraction drugs also requires further investigation.

## Supporting Information

File S1 **Figures S1–S3.** Figure S1: 1A: Correlation between serum bile acid concentration measurements performed on two separate occasions at least 7 days apart (y = 8.1+0.74x, r^2^ = 0.865). 1B: Correlation between serum bile acid concentration and serum concentration of chenodeoxycholic acid (y = 2.7+0.76x, r^2^ = 0.902). 1C: Correlation between tserum bile acid concentration and the sum of the individually determined serum bile acids (y = 7.1+0.99x, r^2^ = 0.930). 1D: Correlation between serum bile acid concentration and serum concentration of cholic acid (y = 0.9+0.20x, r^2^ = 0.889). Figure S2: Effect of (A) intravenous (1 mg) and (B) oral (40 mg) propranolol on blood flow in the superior mesenteric artery (SMA) and portal vein (PV). Blood flow was determined by a Doppler method as described in the text. Figure S3: AUC_0–∞_ after oral dosing (40 mg propranolol) according to (A) serum bile acid concentration and (B) Child class.
(PDF)

File S2 **Tables S1–S2.** Table S1: Maximum change in systolic blood pressure and heart rate (including % change) from baseline after oral propranolol. Table S2: Blood Flow in the Superior Mesenteric Artery (SMA) and Portal Vein (PV) after Intravenous and Oral Propranolol Administration.
(DOCX)
