# Supplementary material for: A Study of the Relationship between Serum Bile Acids and Propranolol Pharmacokinetics and Pharmacodynamics in Patients with Liver Cirrhosis and in Healthy Controls
Source: PLoS One. 2014 Jun 6;9(6):e97885. doi: 10.1371/journal.pone.0097885 (PMC4048194; doi:10.1371/journal.pone.0097885)
Supplement: File S1 — Figures S1–S3. Figure S1: 1A: Correlation between serum bile acid concentration measurements performed on two separate occasions at least 7 days apart (y = 8.1+0.74x, r2 = 0.865). 1B: Correlation between serum bile acid concentration and serum concentration of chenodeoxycholic acid (y = 2.7+0.76x, r2 = 0.902). 1C: Correlation between tserum bile acid concentration and the sum of the individually determined serum bile acids (y = 7.1+0.99x, r2 = 0.930). 1D: Correlation between serum bile acid concentration and serum concentration of cholic acid (y = 0.9+0.20x, r2 = 0.889). Figure S2: Effect of (A) intravenous (1 mg) and (B) oral (40 mg) propranolol on blood flow in the superior mesenteric artery (SMA) and portal vein (PV). Blood flow was determined by a Doppler method as described in the text. Figure S3: AUC0–∞ after oral dosing (40 mg propranolol) according to (A) serum bile acid concentration and (B) Child class. (PDF) [file pone.0097885.s001.pdf]

Suppl Fig. 1

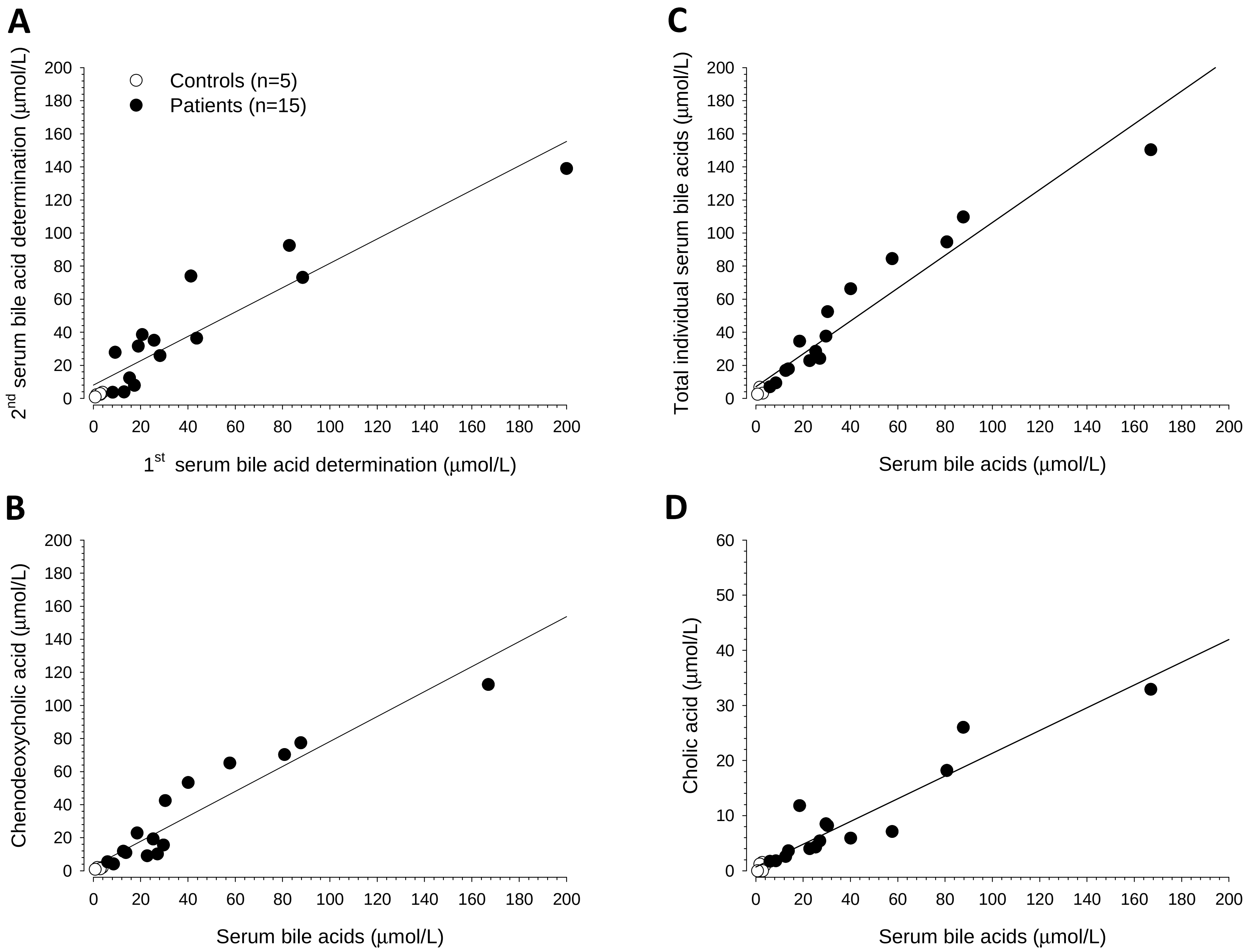

A
Controls (n=5)
Patients (n=15)
2nd serum bile acid determination (µmol/L)
1st serum bile acid determination (µmol/L)
C
Total individual serum bile acids (µmol/L)
Serum bile acids (µmol/L)
B
Chenodeoxycholic acid (µmol/L)
Serum bile acids (µmol/L)
D
Cholic acid (µmol/L)
Serum bile acids (µmol/L)

Suppl Fig. 2

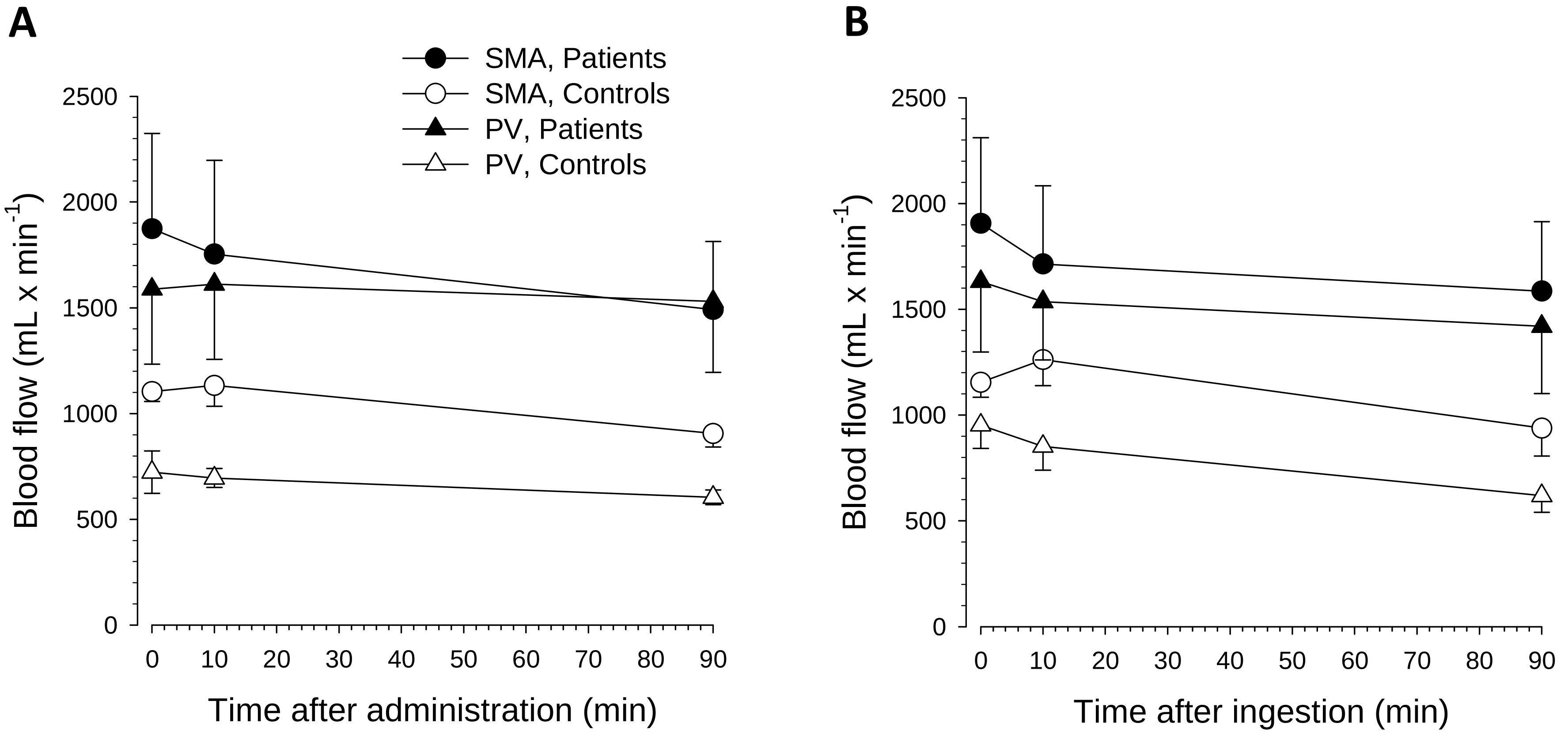
A
B
SMA, Patients
SMA, Controls
PV, Patients
PV, Controls
Blood flow (mL x min-1)
Time after administration (min)
Time after ingestion (min)
0
500
1000
1500
2000
2500
0
10
20
30
40
50
60
70
80
90

Suppl Fig. 3

A

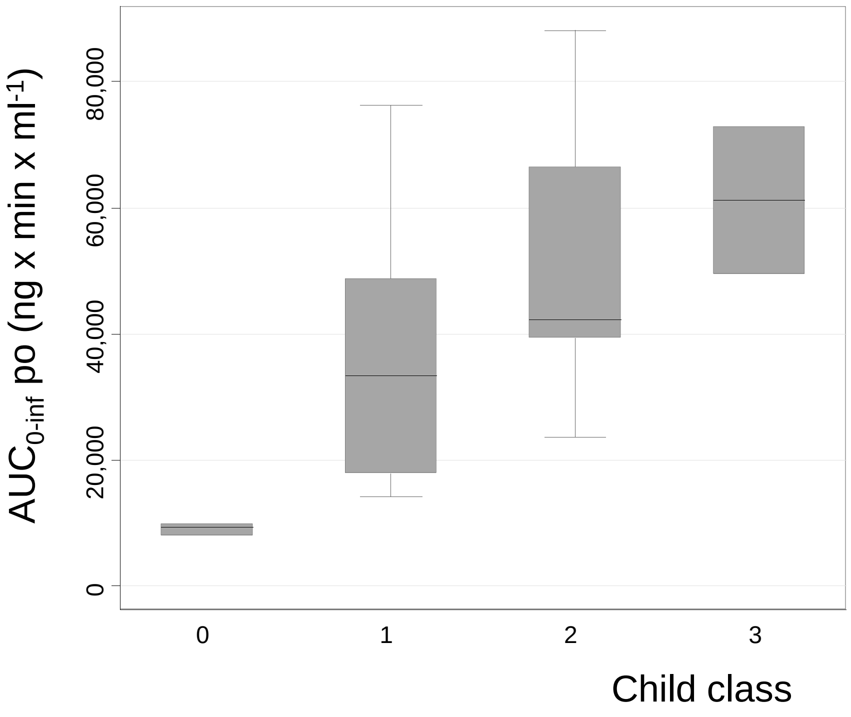

0 = healthy controls (n = 5)
1 = Class A (n = 6)
2 = Class B (n = 5)
3 = Class C (n = 2)

B

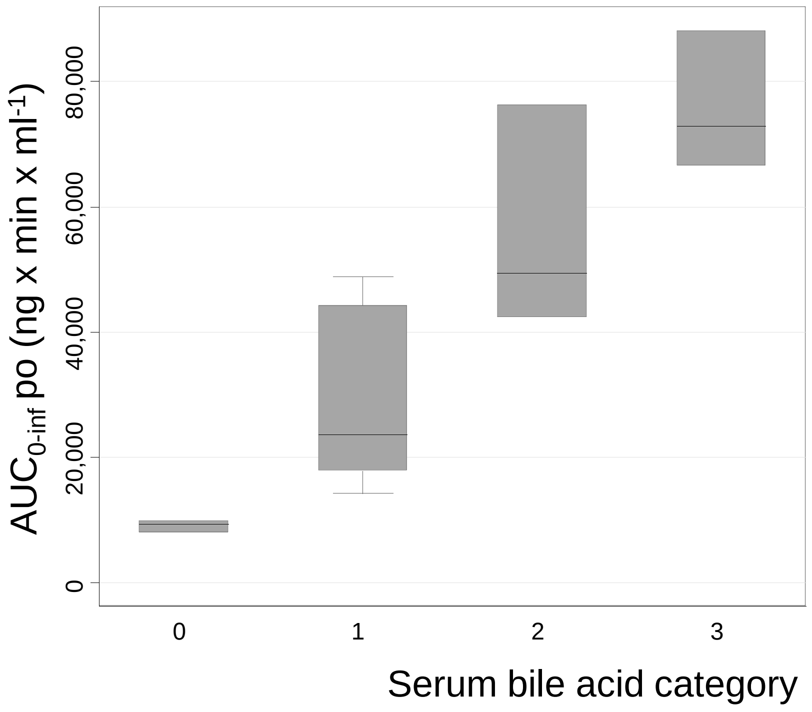

0 = < 6 µmol/l (n = 5 controls)
1 = 6.1 – 20 µmol/l (n = 5 patients)
2 = > 20 - 40 µmol/l (n = 4 patients)
3 = > 40 µmol/l (n = 4 patients)
