# Supplementary material for: A Study of the Relationship between Serum Bile Acids and Propranolol Pharmacokinetics and Pharmacodynamics in Patients with Liver Cirrhosis and in Healthy Controls
Source: PLoS One. 2014 Jun 6;9(6):e97885. doi: 10.1371/journal.pone.0097885 (PMC4048194; doi:10.1371/journal.pone.0097885)
Supplement: File S2 — Tables S1–S2. Table S1: Maximum change in systolic blood pressure and heart rate (including % change) from baseline after oral propranolol. Table S2: Blood Flow in the Superior Mesenteric Artery (SMA) and Portal Vein (PV) after Intravenous and Oral Propranolol Administration. (DOCX) [file pone.0097885.s002.docx]

**A study of the relationship between serum bile acids and propranolol pharmacokinetics and pharmacodynamics in patients with liver cirrhosis and in healthy controls**

Anne B. Taegtmeyer^1*^, Manuel Haschke^1, 2*^, Lydia Tchambaz^1*^, Mirabel Buylaert^1^, Martin Tschöpl^1^, Ulrich Beuers^3^, Jürgen Drewe^1, 2^, Stephan Krähenbühl^1, 2^

*contributed equally to this work

^1^Division of Clinical Pharmacology & Toxicology, University and University Hospital Basel, Basel, Switzerland

^2^Department of Biomedicine, University of Basel, Basel, Switzerland

^3^Department of Gastroenterology & Hepatology, Academic Medical Center, University of Amsterdam, Amsterdam, The Netherlands

**MATERIALS AND METHODS**

**Exclusion criteria**

- Tense ascites
- Encephalopathy grade III-IV
- Persistent alcohol abuse
- Treatment with a beta-blocker
- Concomitant treatment with drugs that inhibit CYP2D6 function (such as amiodarione, celecoxib, cimetidine, clomipramine, fluoxetine, halofantrine, methadone, moclobemide, paroxetine, propafenone, quinidine, ranitidine, ritonavir, terbinafine, thioridacine)
- CYP2D6 poor metabolizer status (as diagnosed by phenotyping using dextrometorphan)
- Actual infection (bacteriemia, spontaneous bacterial peritonitis)
- Cholecystectomy, ileal disease and / or other causes of malabsorption
- Hepatocellular carcinoma
- Heart failure, AV block II or III
- History of asthma or bronchoconstriction

**Pharmacokinetic calculations**

The bioavailability (F) of propranolol was calculated as follows:

(1) $F=\frac{AUC_{0-\infty, oral}\times dose_{iv}}{AUC_{0-\infty, iv}\times dose_{oral}}$

T_max_ and C_max_ values were determined from the raw data. Clearance was estimated using non-linear-regression analysis as follows:

(2) $Cl=\frac{F\times dose}{AUC_{0-\infty}}$

where F is equal to 1 for intravenous administration. The apparent volume of distribution of propranolol was calculated as

(3) $V_{d}=\frac{F\times dose}{AUC_{0-\infty}\times k_{e}}$

where k_e_ is the elimination rate constant. K_e_ was calculated as the slope of the elimination phase after semi logarithmic transformation of the plasma concentration-time curves and was also used to calculate half life T_1/2_ (T_1/2_= ln(2)/k_e_).

**Determination of plasma propranolol concentrations**

The mobile phase (optimized for separation of pronethalol and propranolol) consisted of an aqueous solution of 2.5 mM sodium octanesulfonic acid in phosphoric acid/dihydrogenphosphate 0.01 M (pH 2.5) and acetonitrile (67:33, volume/volume). The flow-rate was kept at 0.8 ml/min and the analysis accomplished in less than 12 minutes. The column effluent was monitored with a fluorescence detector at an excitation wavelength of 230 nm and an emission wavelength of 340 nm. Extraction was performed from 1 ml (intravenous kinetics) or from 0.2 ml human plasma (oral kinetics). As internal standard, 100 μl of an aqueous pronethalol solution (120 ng/ml) was added to 1 ml plasma for iv kinetics and 20 μl of an aqueous pronethalol solution (600 ng/ml) to 200 μl plasma for oral kinetics. After addition of 300 μl (1.4 M) sodium dihydrogencarbonate and vortex mixing for 15 seconds, 9 ml of extraction medium (hexan:ethylacetat 1:1, volume/volume) were added to the sample and the tubes shaken for 20 min. After centrifugation (3000 x g for 5 min), the aqueous layer was frozen and the organic layer recovered and extracted with 300 μl (0.01M) sulfuric acid by shaking the samples for 20 minutes. After centrifugation (3000 x g for 5 min), 250 μl of the aqueous layer were transferred into autosampler vials. An aliquot of 10 μl was injected into the HPLC system. The separation took place in a 3 μm Luna Pheny-Hexyl column (150x4.60 mm internal diameter, Phenomenex, Germany) protected by a phenyl (phenypropyl) guard column (4x3 mm internal diameter, Phenomenex, Germany) and thermostated at 37°C.

The limit of detection for propranolol was 0.15 ng/ml and the limit of quantification 0.5 ng/ml. The mean intra- and interday precision was found to be 2.4% and 7.9%, respectively. The mean inter- and intraday accuracy was 102 and 106%. The analytical recoveries of the drug from heparinized human plasma were determined at four concentrations (48, 13, 6 and 1.5 ng/ml) and ranged from 85.0 to 95.5%.

**Table S1:** Maximum change in systolic blood pressure and heart rate (including % change) from baseline after oral propranolol

| **Subject** | **Maximum change in systolic blood pressure from baseline (mmHg)** | **Maximum change in heart rate from baseline (beats per minute)** | **Maximum percentage change from baseline heart rate (%)** |
| --- | --- | --- | --- |
| Patient 1 | -13 | -34 | -36 |
| Patient 2 | -20 | -14 | -18 |
| Patient 3 | -6 | -9 | -11 |
| Patient 4 | -41 | -29 | -33 |
| Patient 5 | -8 | -10 | -15 |
| Patient 6 | -1 | -10 | -16 |
| Patient 7 | -28 | -8 | -13 |
| Patient 8 | -28 | -3 | -4 |
| Patient 9 | -24 | -9 | -15 |
| Patient 10 | -27 | -12 | -19 |
| Patient 11 | -25 | -17 | -23 |
| Patient 12 | -12 | -22 | -23 |
| Patient 13 | -27 | -15 | -20 |
| Patient 14 | -23 | -15 | -24 |
| Patient 15 | -22 | -14 | -20 |
| Control 1 | -6 | -8 | -17 |
| Control 2 | -22 | -11 | -10 |
| Control 3 | -24 | -2 | -4 |
| Control 4 | -11 | -5 | -7 |
| Control 5 | -8 | -9 | -14 |

**Table S2:** Blood flow in the superior mesenteric artery (SMA) and portal vein (PV) after intravenous and oral propranolol administration.

|  | **Intravenous** | | | | | | | **Oral** | | | | | |
| --- | --- | --- | --- | --- | --- | --- | --- | --- | --- | --- | --- | --- | --- |
|  | **SMA-volume flow (mL/min)** | | | **PV-volume flow (mL/min)** | | | | **SMA-volume flow (mL/min)** | | | **PV-Volume flow (mL/min)** | | |
| **Time (min)** | **0** | **10** | **90** | | **0** | **10** | **90** | **0** | **10** | **90** | **0** | **10** | **90** |
| Patient 2 | 2895 | 2692 | 2201 | | 790 | 825 | 703 | 2879 | 2463 | 2234 | 859 | 870 | 656 |
| Patient 3 | 2360 | 2284 | 1863 | | 1180 | 1137 | 1515 | 2266 | 2228 | 2054 | 1791 | 1664 | 1500 |
| Patient 4 |  |  |  | | 301 | 304 | 301 |  |  |  | 222 | 200 | 114 |
| Patient 5 | 1046 | 775 | 846 | | 2247 | 2172 | 2154 | 1273 | 1158 | 916 | 2275 | 2187 | 1769 |
| Patient 6 | 1194 | 1264 | 1054 | | 1972 | 1865 | 1685 | 1206 | 1007 | 1139 | 1783 | 1629 | 1624 |
| Patient 8 |  |  |  | | 1521 | 1799 | 1528 |  |  |  | 1916 | 1557 | 1588 |
| Patient 10 |  |  |  | | 1154 | 1202 | 983 |  |  |  | 951 | 1109 | 961 |
| Patient 11 |  |  |  | | 3542 | 3587 | 3370 |  |  |  | 3254 | 3071 | 3144 |
| **Mean ± SEM** | 1874±450 | 1754±443 | 1491±323 | | 1588±355 | 1611±355 | 1530±335 | 1906±405 | 1714±369 | 1586±328 | 1631±333 | 1536±305 | 1419±318 |
|  |  |  |  | |  |  |  |  |  |  |  |  |  |
| Control 1 | 1228 | 1402 | 1037 | | 699 | 720 | 621 | 1305 | 1268 | 974 | 718 | 727 | 674 |
| Control 2 | 1018 | 1059 | 801 | | 764 | 732 | 653 | 1006 | 1006 | 685 | 935 | 728 | 618 |
| Control 3 | 1124 | 1130 | 995 | | 471 | 562 | 502 | 1067 | 1595 | 1290 | 908 | 767 | 407 |
| **Mean ± SEM** | 1124±60 | 1197±104 | 944±73 | | 645±89 | 671±55 | 592±46 | 1126±91 | 1289±170 | 983±175 | 853±68 | 741±13 | 566±81 |

**Supporting Information Legends**

File S1 Figure S1

1A: Correlation between serum bile acid concentration measurements performed on two separate occasions at least 7 days apart (y = 8.1 + 0.74x, r^2^ = 0.865).

1B: Correlation between serum bile acid concentration and serum concentration of chenodeoxycholic acid (y = 2.7 + 0.76x, r^2^ = 0.902).

1C: Correlation between tserum bile acid concentration and the sum of the individually determined serum bile acids (y = 7.1 + 0.99x, r^2^ = 0.930).

1D: Correlation between serum bile acid concentration and serum concentration of cholic acid (y = 0.9 + 0.20x, r^2^ = 0.889).

File S1 Figure S2

Effect of (A) intravenous (1 mg) and (B) oral (40 mg) propranolol on blood flow in the superior mesenteric artery (SMA) and portal vein (PV). Blood flow was determined by a Doppler method as described in the text.

File S1 Figure S3

AUC_0- ∞_ after oral dosing (40 mg propranolol) according to (A) serum bile acid concentration and (B) Child class.
